# Optimization and inference of bin widths for histogramming inelastic neutron scattering spectra

**DOI:** 10.1107/S1600576722003624

**Published:** 2022-05-25

**Authors:** Kazuyoshi Tatsumi, Yasuhiro Inamura, Maiko Kofu, Ryoji Kiyanagi, Hideaki Shimazaki

**Affiliations:** a Materials and Life Science Division, J-PARC Center, Japan Atomic Energy Agency, Shirakata 2-4, Tokai, Ibaraki 319-1195, Japan; b Center for Human Nature, Artificial Intelligence and Neuroscience, Hokkaido University, Kita-ku, Sapporo, Hokkaido 060-0812, Japan

**Keywords:** data-driven science, histogram bin-width optimization, inelastic neutron scattering, inhomogeneous Poisson point processes, statistical spectral-feature validation, experiment design

## Abstract

A data-driven bin-width optimization for the histograms of measured data sets based on inhomogeneous Poisson processes was developed in a neurophysiology study [Shimazaki & Shinomoto (2007). *Neural Comput.* **19**, 1503–1527], and a subsequent study [Muto, Sakamoto, Matsuura, Arima & Okada (2019). *J. Phys. Soc. Jpn*, **88**, 044002] proposed its application to inelastic neutron scattering (INS) data. In the present study, the results of the method on experimental INS time-of-flight data collected under different measurement conditions from a copper single crystal are validated. The extrapolation of the statistics on a given data set to other data sets with different total counts precisely infers the optimal bin widths on the latter. The histograms with the optimized bin widths statistically verify two fine-spectral-feature examples in the energy and momentum transfer cross sections: (i) the existence of phonon band gaps; and (ii) the number of plural phonon branches located close to each other. This indicates that the applied method helps in the efficient and rigorous observation of spectral structures important in physics and materials science like novel forms of magnetic excitation and phonon states correlated to thermal conductivities.

## Abstract 2

Bin widths for histograms of inelastic neutron scattering (INS) spectra are optimized for a given data set and inferred for other data sets of different total counts. After verification of the results on INS experimental and simulated data sets, utilization of the method and further improvement are discussed.

## 1. Introduction

In pulsed-neutron experimental facilities, the number of inelastically scattered neutrons collected over a massive 4D space subtended by the momentum transfer **q** and the energy transfer *E* has increased due to the increasing power of spallation sources (Hasegawa *et al.*, 2018 ▸) and novel measurement schemes, such as the continuous rotation of single-crystal samples (Weber *et al.*, 2012 ▸) and multiple incident-neutron energies (Nakamura *et al.*, 2009 ▸). These facilities record each single neutron scattering event (Peterson *et al.*, 2015 ▸); hence, the inelastic neutron scattering (INS) intensity distributions can be flexibly represented without information loss in the most effective form. This is a very interesting aspect of the data stored in these facilities. This characteristic should be utilized by data-driven statistical methods towards a more effective measurement design and a more rigorous analysis of material properties.

The INS intensity distributions are usually represented by histograms in (**q**, *E*). Suitable bin widths are guessed on the basis of knowledge of the instrumental resolution and adopted analysis methods, and are determined by carefully checking whether the results are essentially invariant with the candidates of the bin widths. However, the INS experimental data are statistical variables and the results drawn from them should be statistically verified. One of the possible validations of the results deduced from the histogram is to perform the same experiment twice and compare the results, which requires a doubling of the measurement time. Another is to fit the spectral feature by a valid and more sophisticated analytical function than the histogram, but this may be difficult if the origin of the spectral feature is unclear and the spectral feature is not formulated. Generally, a nonparametric histogram method is suitable for estimating the underlying probability density of such unclarified data (Bishop, 2006 ▸). However, since the choice of a histogram bin width significantly affects the estimation accuracy, it needs to be optimized following a certain statistical criterion. If a set of statistically optimal bin widths is estimated from the given data set, this data-driven estimation can be used to preliminarily and quickly inspect the spectral features in the data set, working to check the INS spectrum at least complementarily to the conventional bin-width selection, because the estimation does not empirically consider the instrumental resolution or the adopted analysis methods.

Shimazaki & Shinomoto (2007 ▸) developed a bin-width optimization method for a time histogram of single neuron firing data in neurophysiology, by assuming that the set of observed neuron firing times was generated by independently repeated trials of inhomogeneous Poisson point processes. Muto *et al.* (2019 ▸) recently extended the method to multi-dimensional data and applied it to simulated INS data sets.

In the approach reported by Shimazaki & Shinomoto (2007 ▸), statistics on a data set were extrapolated to infer the optimal bin widths on a hypothetical data set of a different total trial number from the former data set. By using this extrapolation on INS data, for example, we can infer the optimal bin widths of a future data set from the data set already obtained. If this extrapolation works, and we predict on the fly the necessary total count to reveal the targeted spectral features, we can terminate the INS measurement with sufficient and necessary acquisition time, promoting efficient material analyses. As a data-driven approach, the extrapolation method may be applied for revealing the optimal bin widths of hypothetical data sets even if one does not have prior knowledge of the spectral features. Two possible uses of the inference for cases where the interesting features are unknown are as follows: (i) we can estimate whether continuing the measurement is meaningful by checking if the extrapolated optimal bin widths can be decreased by continuing the measurement within a limited machine time; (ii) we can examine the count dependence of the extrapolated optimal bin widths during the measurement and efficiently terminate the measurement before the optimal bin widths on all of the important axes are unnecessarily smaller than the corresponding point resolutions defined by the measurement condition.

However, the optimization method has not yet been examined on actual experimental INS data. In particular, the inference of the optimal bin widths by extrapolation has not been tried on any experimental and simulated INS data. The standard post-processing in the software packages developed at neutron facilities (Inamura *et al.*, 2013 ▸; Arnold *et al.*, 2014 ▸; Azuah *et al.*, 2009 ▸) applies several corrections to the neutron counts and forms a histogram whose bin heights are proportional to a physically meaningful quantity, such as the scattering law or the double differential scattering cross section (DDSCS) of the INS. We did not apply the corrections and have retained the neutron count statistics during the post-processing, which was necessary for the method utilizing the inhomogeneous Poisson point process (Shimazaki & Shinomoto, 2007 ▸).

Our goal is to utilize the method to perform rigorous and efficient material analyses with INS data. As necessary steps for the final goal, we validate the optimization and extrapolation results on the experimental INS data sets. Moreover, we investigate the uncertainty in the bin-width solutions using simulated data, capturing the major points of the experimental INS data influencing the optimal bin widths.

Machine-learning-based methods have recently been applied to experimental data in large experimental facilities (Hey *et al.*, 2020 ▸), including neutron scattering data (Butler *et al.*, 2021 ▸; Archibald *et al.*, 2020 ▸; Demerdash *et al.*, 2019 ▸; Samarakoon *et al.*, 2020 ▸). The optimization of the histogram representation will provide a basic tool to proceed with machine-learning studies because the optimized histogram shows the right amount of information in the data in the context that it represents the underlying probability density most accurately among histograms with equal bin widths.

## 2. Methodology

### 2.1. Bin-width optimization and extrapolation

Assuming that the INS counts in the 4D space are generated by independently repeated trials of inhomogeneous Poisson point processes, we can optimize the histogram bin widths to minimize the mean integrated squared error (MISE) between the histogram bin heights 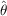 and the underlying Poisson rate 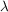 , 

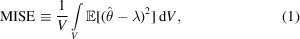

where *V* is the volume of the 4D space and 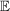 refers to the expectation over different realizations of point events, given 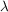 . This is reduced to minimize the following cost function (Shimazaki & Shinomoto, 2007 ▸; Muto *et al.*, 2019 ▸):

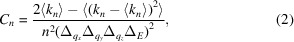




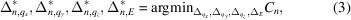

where 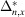 is the optimal bin width along the *x* axis of the histogram containing a total count *n*, 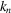 is the neutron count within the bin and 〈·〉 means averaging · over bins. Since we apply the optimization method developed for a point process to density estimation, we use the total count *n* as a pr­oxy of the total trial number of point processes in the original paper. The total count *n* can be replaced with the number of experimental trials if the number of experimental trials performed to obtain the samples is available. The cost function used by Muto *et al.* (2019 ▸) does not contain the factor *n* ^−2^, probably because it is irrelevant for choosing an optimal bin width for the data sets at hand, but inclusion of *n* in the cost function plays a pivotal role for us to infer the optimal bin width of hypothetical data sets of different size as described below.

The cost function can be extrapolated from 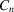 to the one in the hypothetical data containing a total count 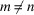 , 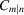 according to the following equation (Shimazaki & Shinomoto, 2007 ▸):

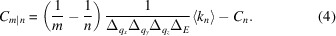

The optimal bin widths can be extrapolated as follows:

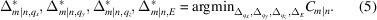




The present input data sets were histograms of fine bin widths. The optimal bin widths were sought within multiples of these initial histogram bin widths. Considering the computational cost, we set the initial bin widths such that the optimized bin widths were two to five times larger than the initial bin widths. We set the initial bin widths by repeating the optimization a couple of times, with different configurations of the initial bin widths. (i) We set the initial bin widths large enough so that the optimization was completed within a small amount of time. (ii) If the optimized bin-width value was 1 in units of the initial bin width, we decreased the initial bin width. If the optimized bin-width value was a number that was too large in units of the initial bin width, we increased the initial bin width. (iii) We performed the optimization with the altered initial bin-width configuration. (iv) We repeated (ii) and (iii) until all of the initial bin widths satisfied the condition that the optimized bin widths were two to five times larger than the initial bin widths. This procedure would require much lower computational cost than if we set the initial bin widths too small.

**Figure 1:**
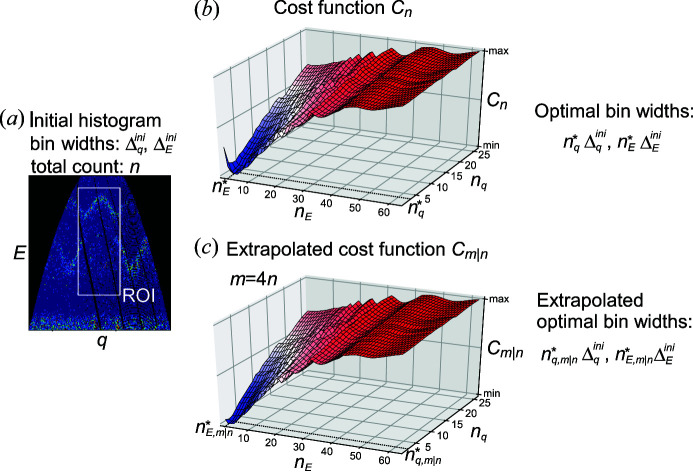
Schematics of the present method for the case of a 2D data set. (*a*) Input of the method as a histogram of fine bin widths 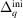 and 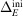 . The method optimizes the bin widths using the count distribution within the ROI. (*b*) Cost function. The bin widths are multiples of 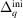 and 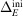 . The integers of multipliers *n_q_* and *n_E_* are shown in the horizontal plane axes. The minimum of the cost function is at (*n_q_* = 2, *n_E_* = 4). (*c*) Cost function extrapolated to a hypothetical data set with a four times larger total count than the actual data set. The minimum of the cost function is at (*n_q_* = 1, *n_E_* = 2).

Fig. 1 ▸ illustrates the procedures using equations (3) and (5). We obtained the optimal bin widths for the intensity distribution within the regions of interest (ROI) formed as a hyper-rectangle in the data space. In order to use the optimization and extrapolation method online with fast enough computation, the bin widths over which we looked for the minimum cost function were limited to be smaller than half of the corresponding edge lengths of the ROI. This limitation may eliminate possible poor solutions where the ROI was represented by a single bin on some axes. This was not the case for our results because the present optimized bin widths were not the upper bounds.

### 2.2. Experimental data

Experimental INS time-of-flight event data were collected on a face-centered cubic (f.c.c.) Cu single crystal with three different measurement conditions; the present method could be powerful on event data, because it enables us to flexibly set the INS histogram bin widths without information losses. Data set 1 was collected at 291 K on J-PARC MLF BL01 4SEASONS (Kajimoto *et al.*, 2011 ▸), while data sets 2 and 3 were collected at 10 K on J-PARC MLF BL14 AMATERAS (Nakajima *et al.*, 2011 ▸). The incident neutron energies *E*_i_ were 50.0, 42.1 and 23.7 meV for data sets 1–3, respectively. The FWHM values at the elastic peak 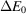 were 4.0, 4.7 and 1.1 meV for data sets 1–3, respectively. To retain the neutron count statistics, we omitted the standard procedures to correct the data and make them proportional to the scattering law, *k*_i_/*k*_f_ correction (*k*_i_ and *k*_f_ are the incoming and outgoing neutron wavenumbers, respectively), averaging counts among the sampling points within a bin, and detector efficiency corrections. One can simply make a histogram of the corrected data with the bin widths optimized on the uncorrected data to obtain an optimal histogram with bin heights proportional to the scattering law.

**Figure 2:**
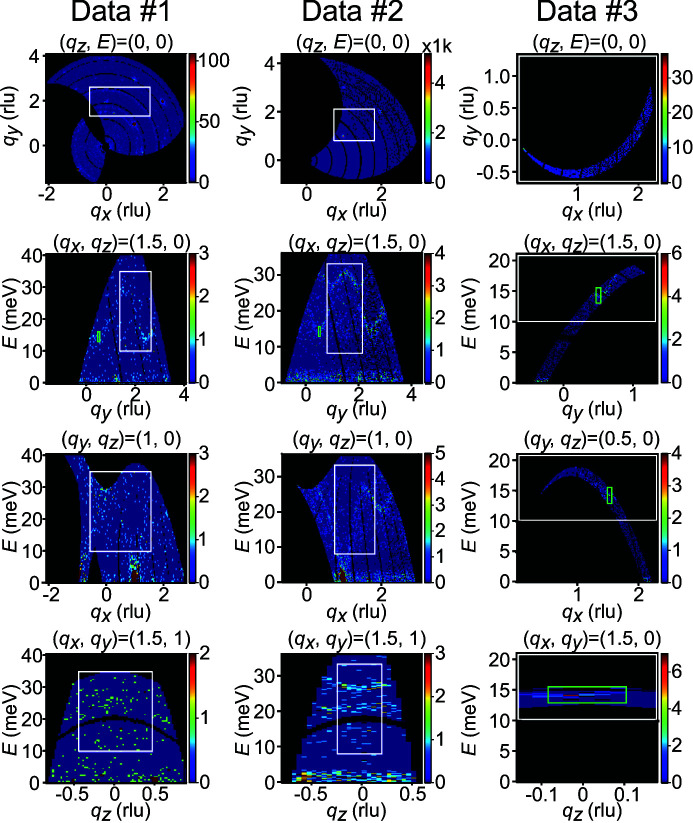
Cross sections of the experimental INS count distributions of the three kinds of data to which we applied the method. 2D slices are taken from the initial histograms; the integration ranges for the other two axes are 1 in units of the initial bin widths. The numerical scales in (**q**, *E*) correspond to the bin centers, except that of *E* in data set 1 corresponds to the bin bottoms. The coordination on the other two axes is shown in units of meV or rlu above each plot. The method optimized the bin widths using the count distribution in a 4D hyper-rectangle located in the white rectangles in the cross sections. The small light-green rectangles in the second row of all data sets and the third and fourth rows of data set 3 show the 4D space commonly included in all data sets. We used the counts within the common 4D space as a measure of the INS intensity of the data.

Fig. 2 ▸ shows cross-sectional views of the initial histograms. The coordination system of **q** is shown in the fourth row of Table 1 ▸. Due to the scattering kinematics and the limited part of the solid angle accessed by the detectors, neutrons cannot be detected in some regions of the 4D space. These masked spaces are colored black. As shown in the fifth row of Table 1 ▸, most of the initial bin widths become finer as the data number increases. The counts in an area common to the three data sets, which is delimited by green rectangles in Fig. 2 ▸, are presented in the sixth row of Table 1 ▸. They increased approximately four times as the data number increased, mainly because the measurement times for a given data space were set longer as the data number increased. We used counts in the common 4D space as a measure of the relative intensities of the data sets.

The white rectangles in Fig. 2 ▸ mark the cross-sectional ROI. These regions are specified in rows seven to 10 of Table 1 ▸. In data sets 1 and 2, we set the ROI as a compromise between excluding the masked space and accumulating the total counts of the unmasked space. Data set 3 had a very narrow unmasked data space along the energy axis, and we allowed a much larger fraction of the masked space than for the other data sets. As will be shown in Section 3.4, the consistency between the experimental energy resolutions and the optimized energy bin widths obtained using the present method partly validated the ROI setting.

The following condition was applied to bin *i* considered in the statistics of 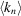 and 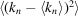 to check the effect of the masked data space on the results:

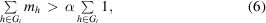

where *h* is the index of the bins in the initial histogram, 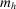 = 0 if the bin is masked (otherwise, 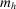 = 1), 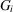 is the set of initial histogram bins included in bin *i*, and α is a constant parameter 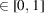 . For data where the unmasked space along a specific axis was very narrow (*e.g.* data set 3), anomalously large bin widths would have the minimum of the cost function for large α because the 4D space considered by the statistics depended on the bin widths through equation (6). We heuristically increased α as much as possible before obtaining the minimum of the cost function located at anomalously large bin widths. The last row of Table 1 ▸ presents the α values.

**Figure 3:**
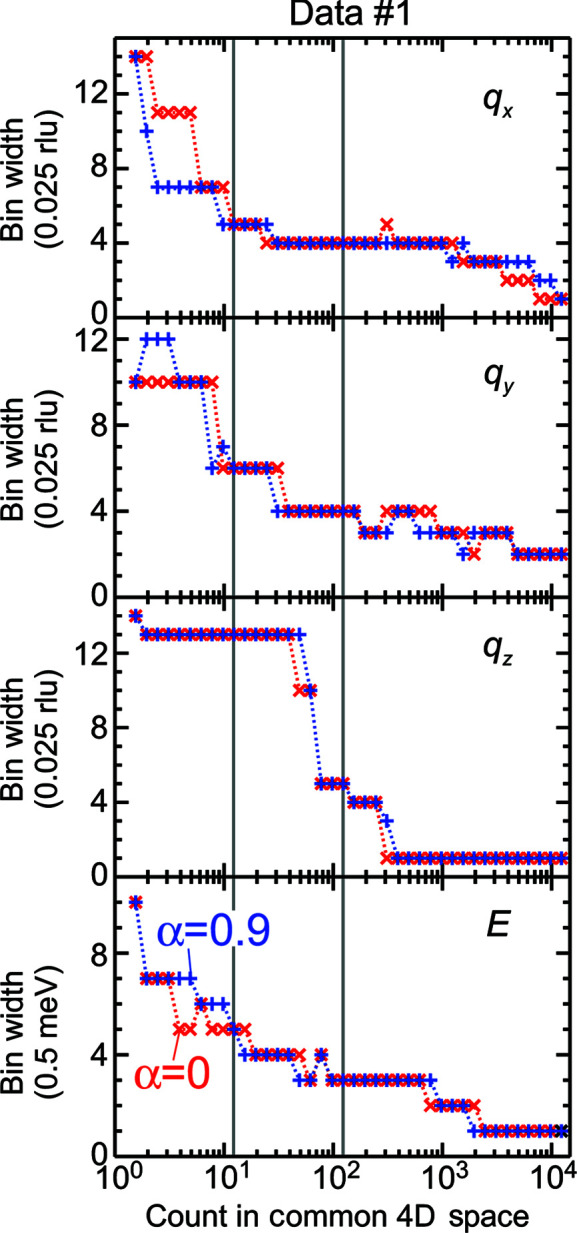
Optimal bin-width results by the extrapolation method for data set 1 with α = 0.9 (blue plus signs) and α = 0 (red crosses), α being a parameter in equation (6); bins with a fraction of masked space larger than this value are taken into account in the calculation of the cost function. The horizontal axis shows the count in the 4D space which is commonly spanned by all of the three kinds of data, located in the green regions in Fig. 2 ▸. The vertical gray lines show the lower and upper bounds of the counts of the corresponding experimental data sets in Section 3.2. The units on the vertical axes are the bin widths of the initial histogram.

Fig. 3 ▸ shows a comparison of the optimal bin widths obtained using equation (5), with α = 0.9 and 0 for data set 1. The count dependencies of the optimal bin widths were very similar between these cases. The same tendency was observed in the other data sets (Fig. S1 in the supporting information). However, we note uncertainties in the optimal bin widths, which are caused by α. They became large in the count region where the optimal bin widths would be altered by small count changes. We expected more stable solutions in the other regions.

### 2.3. Simulated data

The simulated data sets were generated from the theoretical phonon states of f.c.c. Cu calculated within a harmonic phonon approximation in the code *phonopy* (Togo & Tanaka, 2015 ▸) using force constants calculated by the first-principles projector augmented wave method (Kresse & Joubert, 1999 ▸) implemented in the *VASP* software package (Kresse & Furthmüller, 1996 ▸). A quantity proportional to the theoretical DDSCS for the coherent INS was calculated according to the standard formula of Squires (2012 ▸). In the first-principles band calculations of the *VASP* code, the electron–electron interactions were treated by the Perdew–Burke–Ernzerhof density functional within the generalized gradient approximation (GGA-PBE) (Perdew *et al.*, 1996 ▸). The cutoff energy for the plane-wave basis set was 500 eV. For the force constant calculations, a 2 × 2 × 2 supercell of the f.c.c. unit cell was used with an atom in the supercell displaced along [100] by 0.01 Å from the original position. The *k* points for the supercell calculation were on the Monkhorst & Pack (1976 ▸) 3 × 3 × 3 mesh. More details can be found in the supporting information (Section S1).

**Figure 4:**
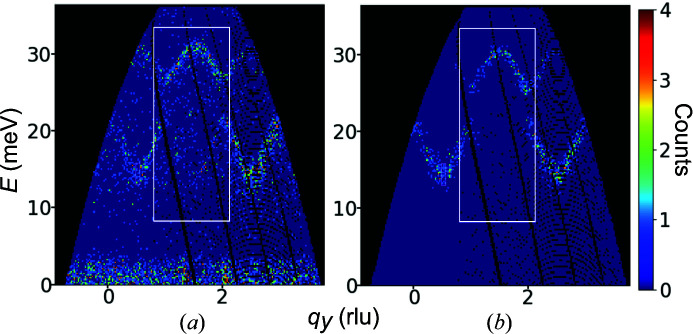
Example cross-sectional views of the (*a*) experimental and (*b*) simulated data set 2 at *q_x_* = 1.5 rlu and *q_z_* = 0 rlu. 2D slices are taken from the initial histograms. The integration ranges for the other two axes and rectangles are the same as in Fig. 2 ▸.

To consider the experimental uncertainty of the counts in (**q**, *E*), the cross section was convoluted with Gaussian functions *N*(*E*) and *N*(**q**), and the neutron counts were randomly generated for each bin under the simulated Poisson rate calculated by integrating the convoluted cross section function over the bin. The initial bin widths and bin locations were the same as in the experimental data sets. The FWHM of *N*(*E*) was varied according to the energy broadening formula (Iida *et al.*, 2014 ▸) with the parameters of the beamlines used for the experimental data. The FWHM of *N*(**q**) was 0.05 Å^−1^, similar to the values reported by Iida *et al.* (2014 ▸). Fig. 4 ▸ shows example cross-sectional views. The count distributions caused by the phonon branches were similar to each other. In contrast, more counts were scattered between the branches in the experimental data, which would be partly reproduced by incorporating the incoherent scattering contribution into the simulated Poisson rate. We considered that the impact of incoherent scattering on the uncertainty of the solutions was small; hence, we neglected it.

### 2.4. Accelerating computation of histograms and cost functions

For a large array of a 4D histogram with a set of small bin widths, speeding up the computation is a prerequisite for the method to be used online during the actual measurement session. This was done in cooperation with a speedy data transformation from the measurement coordinate system to (**q**, *E*), as was done by Shipman *et al.* (2014 ▸) and Inamura *et al.* (2018 ▸). We accelerated the cost function calculation using the cumulative sums of the neutron counts to form more than 10^6^ histograms in a manner similar to that of Muto *et al.* (2019 ▸). The actual formulas are described in Section S2. We implemented the calculation of the histograms and the cost functions as a parallelized Fortran library with a message passing interface library (https://www.open-mpi.org/). Using a 32 core Xeon processor, the bin-width optimization for a single data set lasted for about 20 s for data sets 1 and 2, and about 4 min for data set 3.

## 3. Results and discussion

### 3.1. Energy resolution and optimal energy bin widths

As a first step in verification of the present results of the method, Table 2 ▸ shows the optimized energy bin widths using equation (3) and the FWHM of the energy peak at a phonon band energy minimum at point *L*, (*q_x_*, *q_y_*, *q_z_*, *E*) = (1.5 rlu, *n* + 0.5 rlu, 0 rlu, 15 meV), where *n* = 2, 1 and 0 for data sets 1–3, respectively. We regarded the FWHM values as a measure of the energy resolution in the experimental data sets. Both were obtained from the experimental data sets formed by the event data over the whole measurement times. The optimal bin widths were not much larger than the FWHM values. The present optimization results did not contradict the energy resolution estimation using the FWHM values, partly validating the present optimization results.

We mention for clarity that the optimal bin widths in (**q**, *E*) need not be close to the instrumental resolution. The optimal bin-width values depend on the statistical quality of the given data set. The optimal bin widths of a data set containing sharp features and having a large total count, for example, would be smaller than the instrumental resolution because λ varies greatly with respect to (**q**, *E*) and its MISE in equation (1) will be decreased for a data set with a set of smaller bin widths. This argument is consistent with Fig. 3 ▸, where the optimal energy bin width is decreased as the count is increased beyond the value corresponding to the experimental total measurement time.

### 3.2. Consistency between the optimization and extrapolation results

**Figure 5:**
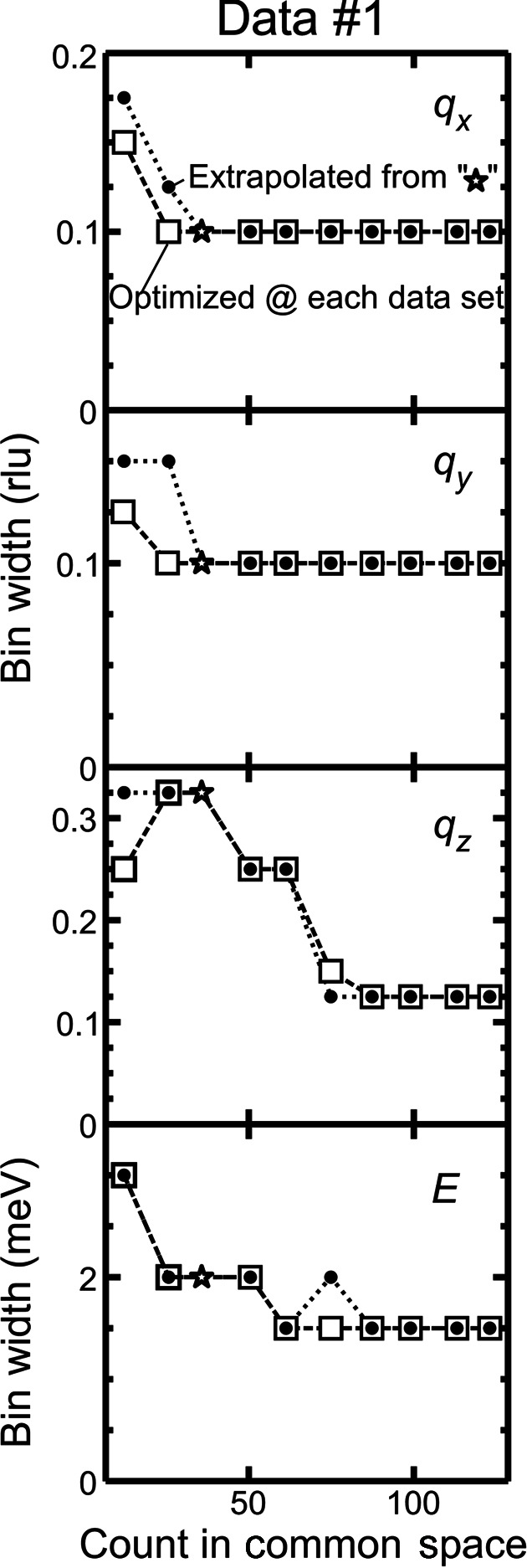
Comparison of the optimal bin-width results by the optimization on the actual data sets with different total counts (squares) and by the extrapolation of the statistics from a single data set (star) to the hypothetical data sets with different total counts (dots). The results obtained are for data set 1. The minimum tick spacings on the vertical axis are the bin widths of the initial histogram.

Several data sets of different total counts were prepared from different parts of the event data by taking only the events from the initial time to different final times. Using the extrapolation method of equation (5) on a data set of a total count *n*, we inferred the optimal bin widths on the data sets of different total counts *m*. Fig. 5 ▸ shows these bin widths with those optimized according to equation (3) on the actual data sets of the total counts *m* for data set 1. The results on the data set of the total count *n* are denoted by stars. We extrapolated the bin widths to smaller count data sets as well in order to see the trend over the whole experimental count range. The extrapolated bin widths were mostly in line with the optimized ones. This was the case for data sets 2 and 3 (Fig. S2). Therefore, the extrapolation method was demonstrated to predict the optimal bin widths for the data sets of prolonged measurement times, which could be used to determine the necessary total count to reveal the targeted spectral features.

The use of the total count numbers for *n* and *m*, instead of the use of the experimental trial numbers for *n* and *m*, works on the present data sets because of the above consistency. An experimental trial could be an INS acquisition sequence for which the whole range of the sample crystal rotation angles from the predefined starting angle to the predefined ending angle is swept once. The total sequence numbers for *n* and *m* appear to be easy to use online, and this approach also rigorously follows the original paper as mentioned in Section 2.1.

In Fig. 5 ▸, the extrapolated energy bin width shows an anomalous jump upwards at count 75: the *q*_*z*_ bin width decreases by 7 initial bin widths while the energy bin width increases by 1 from count 61 to 75. The tiny jump compared with the decrement in the other axis can happen because the cost function contains statistical noise and/or because we searched for the cost function minimum in the limited search space in the bin widths, *i.e.* multiples of the initial bin widths. This problem may be solved by more efficient optimization, for example, Gaussian process regression (Rasmussen & Williams, 2006 ▸), to search for the cost function minimum.

### 3.3. General count dependencies of the optimal bin widths on the experimental data sets

Shimazaki & Shinomoto (2007 ▸), assuming specific functional forms for the autocorrelation of the Poisson rate, derived the following relationship between the total counts *m* and the optimal bin widths 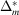 for the 1D histograms:

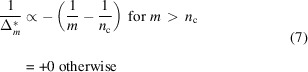

where 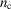 is a constant determined by the Poisson rate and the autocorrelation function.

**Figure 6:**
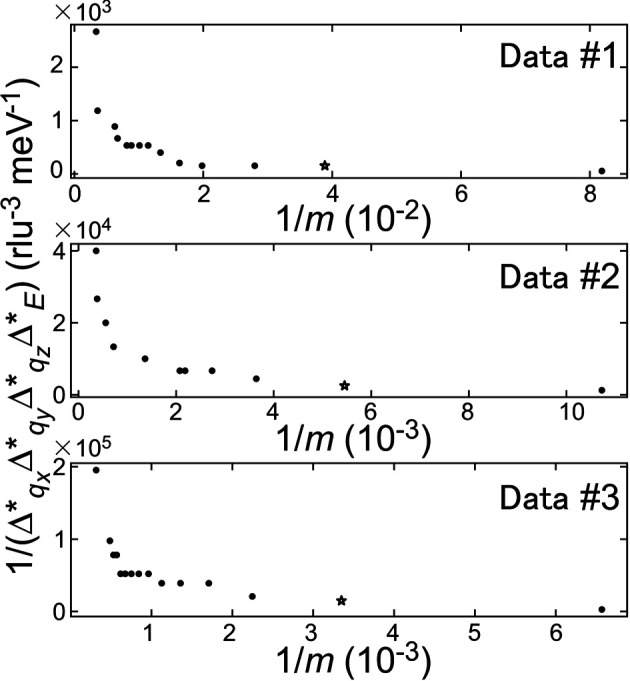
Relationship between the inverses of the optimal bin-width product and the total count numbers. The optimal bin widths are obtained by the extrapolation from the statistics on the experimental data sets corresponding to the results shown as stars.

Fig. 6 ▸ plots the inverse of the optimal bin-width products 1/ 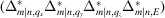 with the inverse of the total neutron counts 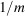 . For a large 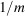 , 1/ 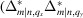 × 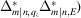 slightly increased as 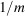 decreased. In contrast, for a small 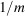 , 1/ 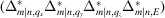 noticeably increased with a decrease in 1/*m*. This behavior was qualitatively consistent with equation (7), partly validating the results.

### 3.4. Count dependencies of the optimal bin widths on the simulated and experimental data sets

**Figure 7:**
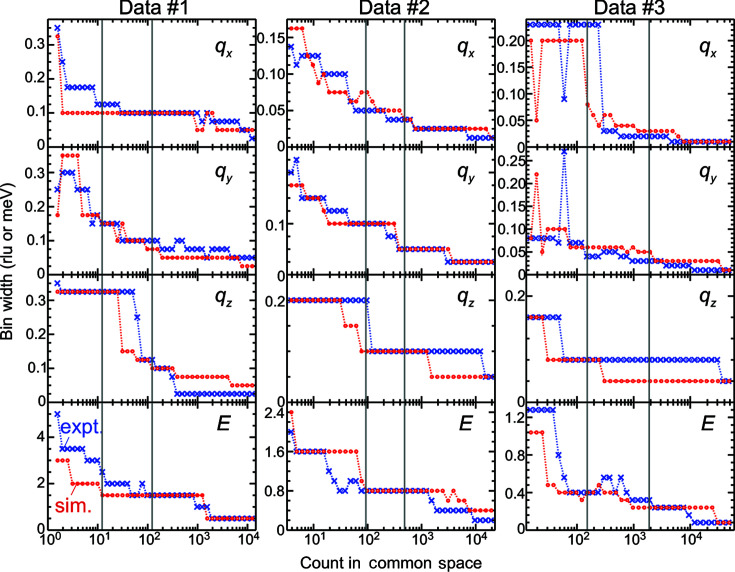
Count dependencies of the optimal bin widths extrapolated from the statistics on the experimental (blue crosses) and the simulated data sets (red circles). The vertical gray lines show the lower and upper bounds of the counts of the corresponding experimental data sets in Section 3.2. The minimum tick spacings on the vertical axis are the bin widths of the initial histogram.

**Figure 8:**
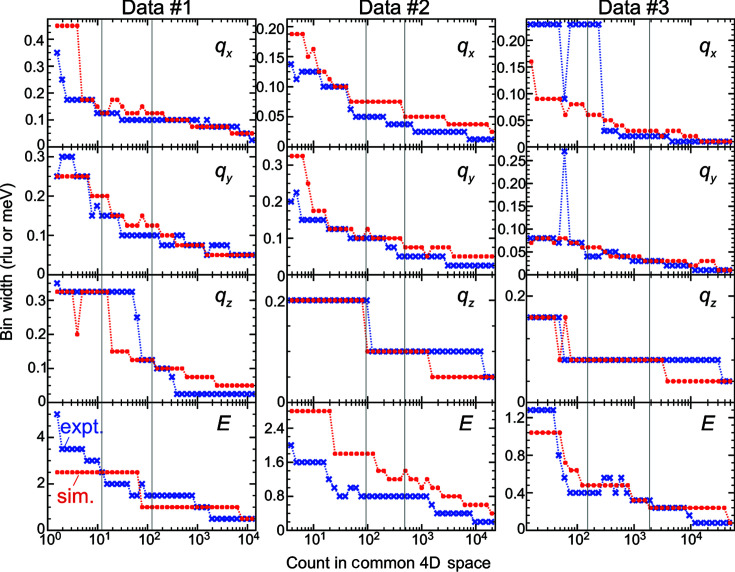
Comparison of the count dependencies of the optimal bin widths extrapolated from the statistics on the experimental (blue crosses) and simply simulated data sets (red circles). Refer to the details in the main text. The vertical gray lines and the minimum tick spacings on the vertical axis are the same as in Fig. 7 ▸.

Fig. 7 ▸ presents a comparison of the extrapolation results on the experimental and simulated data sets. The count dependencies of the bin widths in each panel were approximately consistent between the experimental and simulated data. Therefore, the simulated data represented the major points of the experimental data determining the optimal bin widths. On the basis of this consistency, we will investigate the uncertainty in the solution of the method in Section 3.5. In the work of Muto *et al.* (2019 ▸), the simulated INS data were generated from a Poisson rate synthesized simply by convoluting a Lorentzian energy function of a fixed width with phonon branches. We tested the method on simulated data similar to those used by Muto *et al.* (2019 ▸), generated from a Poisson rate synthesized by a Lorentzian energy function of 3.2 meV FWHM with phonon branches. The optimal bin widths for the more simply simulated data sets 1 and 2 showed larger discrepancies from the optimal bin widths on the experimental data (Fig. 8 ▸). This was not the case for data set 3, partly because the α values of the experimental and simulated data shown in Table 1 ▸ differed. Thus, we preferred applying the method on more rigorously simulated data sets to investigate the uncertainty of the solution.

### 3.5. Uncertainty of solutions

**Figure 9:**
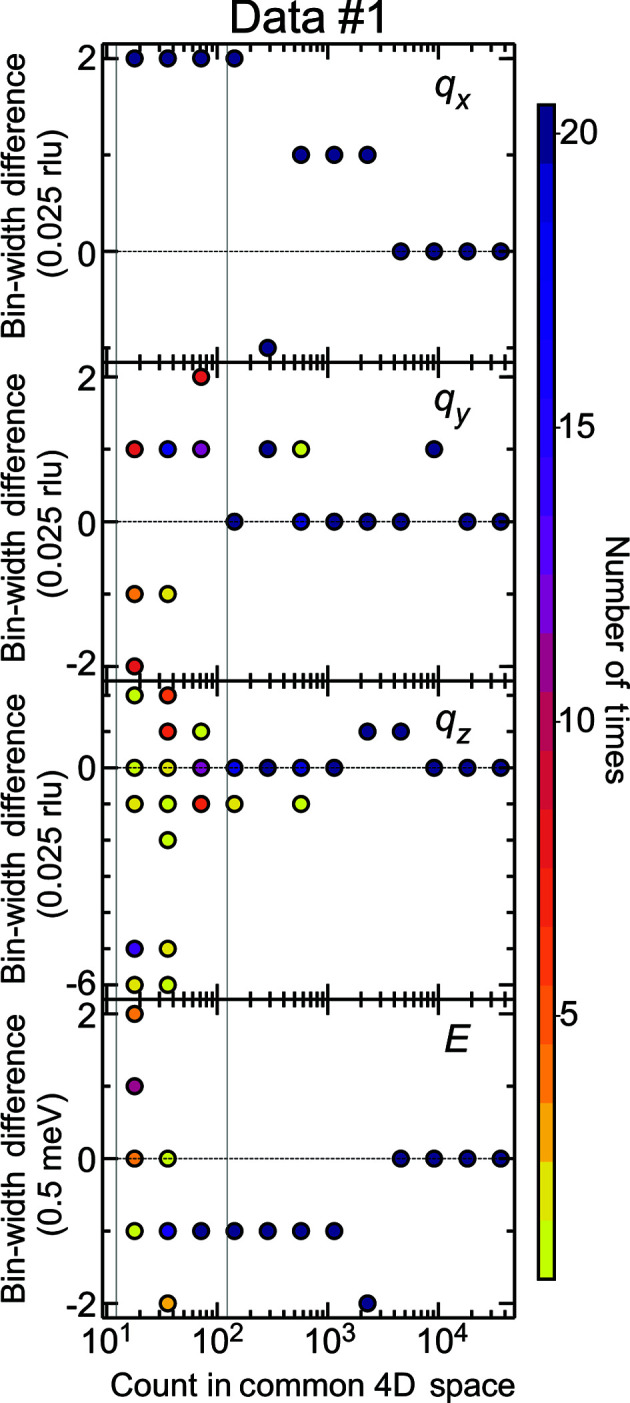
Convergence of the bin-width solution with respect to the count numbers for the simulated data set 1. The optimization method using equation (3) is applied on the simulated data. The differences in the optimized bin widths from the bin widths minimizing the integrated squared errors are plotted for the 20 data sets for each total count number. The color scale indicates the number of times the same differences were obtained in 20 trials. The vertical gray lines show the lower and upper bounds of the counts of the corresponding experimental data sets in Section 3.2. The units on the vertical axes are the bin widths of the initial histogram.

For a simulated data set, we could find a set of bin widths 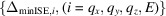 , which, among the configurations of the multiples of the initial bin widths, had the smallest integrated squared error between the simulated Poisson rate and the corresponding histogram. We considered 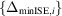 as the ‘true’ solution and investigated the difference between 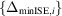 and the set of bin widths optimized using equation (3). Fig. 9 ▸ depicts the results for the simulated data set 1, where 20 sets of the simulated data for each total count number were tried. The number of times the same difference was obtained for the 20 trials is represented by the colors in each circular point. The minimum tick spacing on the vertical axis corresponds to the bin width of the initial histogram. The vertical gray lines denote the upper and lower bounds of the counts considered for the corresponding experimental data sets in Section 3.2. At counts equal to or more than the upper bound, the bin-width differences were converged within two initial histogram bin widths. This was the case for the convergence in data sets 2 and 3 (Fig. S4). Therefore, the uncertainty of the solutions due to the statistics of the cost function with the same mask treatment was estimated as two in units of the bin widths of the initial histogram. If the initial bin widths are finer, the uncertainty will be estimated more accurately, but we did not use the finer initial bin widths because the present accuracy on the uncertainty is enough for the validation of the present method.

### 3.6. Comparison of the optimized histograms between data sets of different qualities

**Figure 10:**
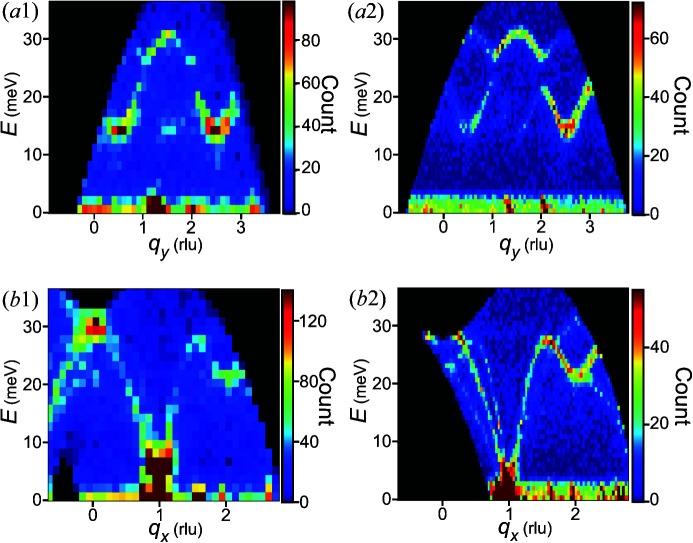
Comparison of the histograms of the optimized bin widths between the experimental data sets of different qualities: (*a*1), (*a*2) cross-sectional views on *q_x_* = 1.5 rlu and *q_z_* = 0 rlu; and (*b*1), (*b*2) *q_y_* = 1.0 rlu and *q_z_* = 0 rlu. Numbers 1 and 2 are for data sets 1 and 2, respectively.

The 4D spaces of data sets 1 and 2 were significantly overlapped; thus, we can compare the cross-sectional views of the histograms with optimized bin widths between these data sets, whose qualities were different, as seen from the count numbers in the sixth row of Table 1 ▸. In Fig. 10 ▸(*a*1) for the lower-quality data, we cannot find a gap at *q_y_* = 1 rlu and *E* = 25 meV. In contrast, a gap exists in Fig. 10 ▸(*a*2) for the higher-quality data. Similarly, Fig. 10 ▸(*b*1) does not clearly show three branches around *q_x_* = 0 to 1 rlu and *E* = 10 to 20 meV, whereas Fig. 10 ▸(*b*2) illustrates them.

These contrasts demonstrate that, by using statistically optimal bin widths, we can objectively and rigorously examine the existence of faint structures in the data set whose optimal bin widths are analyzed. Triplon band splitting was recently observed by INS on the spin-1/2 2D dimerized antiferromagnet Ba_2_CuSi_2_O_6_Cl_2_ (Nawa *et al.*, 2019 ▸). Due to the two dimensionality of the INS distribution in this system, band splitting was clearly demonstrated by integrating the INS intensity along with the axis along which the spectrum was dispersion-less. Selecting adequate bin widths and data statistics was important in seeing the band splitting details. This is the case for the fine structures of the phonon branches associated with the cation order/disorder phases of AgBiSe_2_ (Niedziela *et al.*, 2020 ▸) and the fragmental phonon branches of the isolated point-defect-like intrinsic localized mode suggested in PbSe (Manley *et al.*, 2019 ▸). In general, interesting but faint signals of the INS of materials studied in physics and materials science could be seen by efficiently capturing counts according to the optimal bin widths inferred by the extrapolation method [*e.g.* the abovementioned triplon, magnetic excitations of the frustrated system (Ito *et al.*, 2017 ▸), and phonon states correlated to thermal conductivities (Niedziela *et al.*, 2020 ▸; Manley *et al.*, 2019 ▸; Kajimoto *et al.*, 2018 ▸; Wu *et al.*, 2020 ▸)].

We add a few words on the limitations of the present method. First, if we extrapolate the cost function from the one obtained from small samples, the method suffers considerable sample noise. We can determine whether we have enough samples by checking if the optimal bin width of the current data set is smaller than the observation range of the data. We have already mentioned this check at the end of Section 2.2. Second, we were not able to accurately quantify the sizes of the spectral features (*e.g.* line widths of the phonon branches) from the histogram with the optimized bin widths because of the poor fit of the bars in the histograms to the underlying probability densities. Alternative methods based on adaptive kernels seem promising because they more accurately represent the probability densities of the INS (Shimazaki & Shinomoto, 2010 ▸). However, estimating the density with histogram bins or kernel bandwidths that were adaptively and locally optimized at every position, as done for 1D cases (Shimazaki & Shinomoto, 2010 ▸; Endres & Földiák, 2005 ▸), will be more challenging for higher dimensions. For such data, one may combine the copula method (Trivedi & Zimmer, 2007 ▸) with the 1D adaptive kernel method to estimate the density. The copula allows us to represent the joint density as a product of the 1D marginal distributions and their dependency (copula density). Therefore, one can apply the 1D adaptive kernel method to estimate the marginal distributions separately. At the same time, one can perform a non­parametric histogram or a kernel density estimation of the multi-dimensional copula density while optimizing a single bin or a bandwidth common across the dimensions because the copula density is defined on uniform marginal distributions. Such an approach can potentially improve the accuracy and reduce the computational cost for estimating the multi-dimensional density.

## 4. Summary

In this study, we applied the bin-width optimization and extrapolation method on experimental INS data. The main results are summarized as follows:

(i) We found no contradiction between the optimized energy bin widths and the experimental energy resolutions for three kinds of experimental data collected from the same sample but with different measurement conditions.

(ii) The optimal bin widths obtained by the extrapolation method precisely inferred the optimized bin widths on the actual data sets. Therefore, the extrapolation method is promising for the determination of the necessary total count to reveal the targeted spectral structure and the appropriate measurement termination.

(iii) The relationship between the inverse of the optimal bin-width product and the inverse of the count is consistent with the equation derived for the 1D data by Shimazaki & Shinomoto (2007 ▸).

(iv) Using the simulated data, we speculated that the uncertainty in the optimized bin-width solutions caused by the statistics of the cost function with the same mask treatment was within two in units of the initial histogram bin widths.

(v) For data sets of different qualities, the histograms with the optimized bin widths clarified the existence of fine structures of a phonon band gap and the number of adjacent phonon branches in the INS spectra, demonstrating that, under the condition of having the corresponding data, the histogram with the optimized bin widths could be used to objectively and rigorously examine the fine structures studied in physics and materials science (*e.g.* magnetic triplon bands, magnetic excitations of a frustrated system and phonon states correlated to thermal conductivities).

For the method to be utilized in INS experiments and analyses, more interest must come from the users of neutron experimental facilities. We expect this paper to convey the method to these users and promote the efficiency of material analyses through neutron scattering experiments. To help the method demonstrate its impact at research facilities, we intend to make the code open and easy to use by visiting researchers.

## Supplementary Material

Details of the preparation of simulated data, and formulas for calculating 4D histograms using cumulative sums, Figs. S1, 2, 3 on data sets 2 and 3, corresponding to Figs. 3, 5 and 9 on data set 1. DOI: 10.1107/S1600576722003624/in5064sup1.pdf

## Figures and Tables

**Table 1:** Specifications of the three kinds of experimental data α in the last row is a parameter in equation (6); bins with a fraction of the masked space larger than this value are taken into account in the calculation of the cost function.

Data set	1	2	3
Incident neutron energy, *E*_i_ (meV)	50.0	42.1	23.7
FWHM @ elastic, 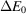 (meV)	4.0	4.7	1.1
Directions of *q_x_*, *q_y_*, *q_z_* †	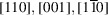		
Initial bin widths, 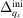 , 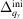 , 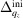 , 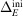 ‡	0.025, 0.025, 0.025, 0.5	0.0125, 0.025, 0.05, 0.2	0.01, 0.01, 0.04, 0.08
Count in a common 4D space§	123	483	1885
Analyzed data area‡	−0.575 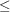 *q_x_* 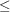 1.525	0.75 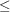 *q_x_* 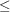 1.825	0.01 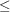 *q_x_* 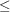 2.29
	1.35 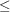 *q_y_* 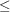 2.65	0.80 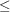 *q_y_* 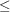 2.125	−0.67 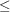 *q_y_* 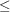 1.35
	−0.45 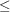 *q_z_* 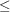 0.475	−0.25 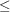 *q_z_* 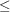 0.20	−0.16 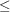 *q_z_* 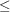 0.18
	10 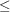 *E* 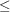 35	8.2 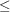 *E* 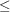 33.4	10.0 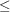 *E* 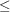 21.12
α	0.9	0.9	0.7¶, 0.5††

† In conventional reciprocal-lattice units (rlu).

‡ In rlu and meV for the reciprocal and energy coordinates, respectively.

§ Acquired in the whole measurement time within the data space of 0.475 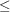 *q_x_* 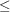 0.525 rlu, 1.475 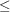 *q* 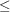 1.525 rlu, −0.1 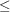 *q_z_* 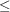 0.1 rlu and 13.0 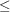 *E* 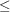 15.5 meV.

¶ For the experimental data.

†† For the simulated data.

**Table 2:** FWHM (meV) of the INS peak at the *L* point [*q_x_ q_y_ q_z_* (rlu), *E* (meV)] and optimized energy bin widths (meV)

Data set	(*q_x_ q_y_ q_z_*, *E*)	FWHM	Δ^*^ *_E_*
1	(1.5 2.5 0, 15)	3	1.5
2	(1.5 1.5 0, 15)	3	0.8
3	(1.5 0.5 0, 15)	0.5	0.32

## References

[bb1] Archibald, R. K., Doucet, M., Johnston, T., Young, S. R., Yang, E. & Heller, W. T. (2020). *J. Appl. Cryst.* **53**, 326–334.

[bb3] Arnold, O., Bilheux, J. C., Borreguero, J. M., Buts, A., Campbell, S. I., Chapon, L., Doucet, M., Draper, N., Ferraz Leal, R., Gigg, M. A., Lynch, V. E., Markvardsen, A., Mikkelson, D. J., Mikkelson, R. L., Miller, R., Palmen, K., Parker, P., Passos, G., Perring, T. G., Peterson, P. F., Ren, S., Reuter, M. A., Savici, A. T., Taylor, J. W., Taylor, R. J., Tolchenov, R., Zhou, W. & Zikovsky, J. (2014). *Nucl. Instrum. Methods Phys. Res. A*, **764**, 156–166.

[bb4] Azuah, R. T., Kneller, L. R., Qiu, Y., Tregenna-Piggott, P. L. W., Brown, C. M., Copley, J. R. D. & Dimeo, R. M. (2009). *J. Res. Natl Inst. Stand. Technol.* **114**, 341–358. 10.6028/jres.114.025 PMC4646530 27504233

[bb5] Bishop, C. M. (2006). *Pattern Recognition and Machine Learning.* Singapore: Springer Science+Business Media.

[bb6] Butler, K. T., Le, M. D., Thiyagalingam, J. & Perring, T. G. (2021). *J. Phys. Condens. Matter*, **33**, 194006. 10.1088/1361-648X/abea1c 33635282

[bb7] Demerdash, O., Shrestha, U. R., Petridis, L., Smith, J. C., Mitchell, J. C. & Ramanathan, A. (2019). *Front. Mol. Biosci.* **6**, 64. 10.3389/fmolb.2019.00064 PMC6705226 31475155

[bb8] Endres, D. & Földiák, P. (2005). *IEEE Trans. Inf. Theory*, **51**, 3766–3779.

[bb9] Hasegawa, K., Hayashi, N., Oguri, H., Yamamoto, K., Kinsho, M. & Yamazaki, Y. (2018). *Proceedings of IPAC2018*, pp. 1038–1040. Geneva: JACoW Publishing.

[bb10] Hey, T., Butler, K., Jackson, S. & Thiyagalingam, J. (2020). *Philos. Trans. R. Soc. A*, **378**, 20190054. 10.1098/rsta.2019.0054 PMC7015290 31955675

[bb11] Iida, K., Ikeuchi, K., Ishikado, M., Suzuki, J., Kajimoto, R., Nakamura, M., Inamura, Y. & Arai, M. (2014). *JPS Conf. Proc.* **1**, 014016.

[bb12] Inamura, Y., Ito, T. & Suzuki, J. (2018). *J. Phys. Conf. Ser.* **1021**, 012015.

[bb13] Inamura, Y., Nakatani, T., Suzuki, J. & Otomo, T. (2013). *J. Phys. Soc. Jpn*, **82**, SA031.

[bb14] Ito, S., Kurita, N., Tanaka, H., Ohira-Kawamura, S., Nakajima, K., Itoh, S., Kuwahara, K. & Kakurai, K. (2017). *Nat. Commun.* **8**, 235. 10.1038/s41467-017-00316-x PMC5550445 28794443

[bb15] Kajimoto, R., Nakamura, M., Inamura, Y., Mizuno, F., Nakajima, K., Ohira-Kawamura, S., Yokoo, T., Nakatani, T., Maruyama, R., Soyama, K., Shibata, K., Suzuya, K., Sato, S., Aizawa, K., Arai, M., Wakimoto, S., Ishikado, M., Shamoto, S., Fujita, M., Hiraka, H., Ohoyama, K., Yamada, K. & Lee, C.-H. (2011). *J. Phys. Soc. Jpn*, **80**, SB025.

[bb16] Kajimoto, R., Nakamura, M., Murai, N., Shamoto, S., Honda, T., Ikeda, K., Otomo, T., Hata, H., Eto, T., Noda, M., Kuwahara, H. & Okuda, T. (2018). *Sci. Rep.* **8**, 9651. 10.1038/s41598-018-27984-z PMC6018226 29941897

[bb17] Kresse, G. & Furthmüller, J. (1996). *Phys. Rev. B*, **54**, 11169–11186. 10.1103/physrevb.54.11169 9984901

[bb18] Kresse, G. & Joubert, D. (1999). *Phys. Rev. B*, **59**, 1758–1775.

[bb19] Manley, M. E., Hellman, O., Shulumba, N., May, A. F., Stonaha, P. J., Lynn, J. W., Garlea, V. O., Alatas, A., Hermann, R. P., Budai, J. D., Wang, H., Sales, B. C. & Minnich, A. J. (2019). *Nat. Commun.* **10**, 1928. 10.1038/s41467-019-09921-4 PMC6486597 31028271

[bb20] Monkhorst, H. J. & Pack, J. D. (1976). *Phys. Rev. B*, **13**, 5188–5192.

[bb21] Muto, K., Sakamoto, H., Matsuura, K., Arima, T. & Okada, M. (2019). *J. Phys. Soc. Jpn*, **88**, 044002.

[bb22] Nakajima, K., Ohira-Kawamura, S., Kikuchi, T., Nakamura, M., Kajimoto, R., Inamura, Y., Takahashi, N., Aizawa, K., Suzuya, K. K., Shibata, K. T., Nakatani, T., Soyama, K., Maruyama, R., Tanaka, H., Kambara, W., Iwahashi, T., Itoh, Y., Osakabe, T., Wakimoto, S., Kakurai, K., Maekawa, F., Harada, M., Oikawa, K., Lechner, R. E., Mezei, F. & Arai, M. (2011). *J. Phys. Soc. Jpn*, **80**, SB028.

[bb23] Nakamura, M., Kajimoto, R., Inamura, Y., Mizuno, F., Fujita, M., Yokoo, T. & Arai, M. (2009). *J. Phys. Soc. Jpn*, **78**, 093002.

[bb24] Nawa, K., Tanaka, K., Kurita, N., Sato, T. J., Sugiyama, H., Uekusa, H., Ohira-Kawamura, S., Nakajima, K. & Tanaka, H. (2019). *Nat. Commun.* **10**, 2096. 10.1038/s41467-019-10091-6 PMC6506493 31068576

[bb25] Niedziela, J. L., Bansal, D., Ding, J., Lanigan-Atkins, T., Li, C., May, A. F., Wang, H., Lin, J. Y. Y., Abernathy, D. L., Ehlers, G., Huq, A., Parshall, D., Lynn, J. W. & Delaire, O. (2020). *Phys. Rev. Mater.* **4**, 105402.

[bb26] Perdew, J. P., Burke, K. & Ernzerhof, M. (1996). *Phys. Rev. Lett.* **77**, 3865–3868. 10.1103/PhysRevLett.77.3865 10062328

[bb27] Peterson, P. F., Campbell, S. I., Reuter, M. A., Taylor, R. J. & Zikovsky, J. (2015). *Nucl. Instrum. Methods Phys. Res. A*, **803**, 24–28.

[bb28] Rasmussen, C. E. & Williams, C. K. I. (2006). *Gaussian Processes for Machine Learning.* Cambridge: The MIT Press.

[bb29] Samarakoon, A. M., Barros, K., Li, Y. W., Eisenbach, M., Zhang, Q., Ye, F., Sharma, V., Dun, Z. L., Zhou, H., Grigera, S. A., Batista, C. D. & Tennant, D. A. (2020). *Nat. Commun.* **11**, 892. 10.1038/s41467-020-14660-y PMC7021707 32060263

[bb30] Shimazaki, H. & Shinomoto, S. (2007). *Neural Comput.* **19**, 1503–1527. 10.1162/neco.2007.19.6.1503 17444758

[bb31] Shimazaki, H. & Shinomoto, S. (2010). *J. Comput. Neurosci.* **29**, 171–182. 10.1007/s10827-009-0180-4 PMC2940025 19655238

[bb32] Shipman, G., Campbell, S., Dillow, D., Doucet, M., Kohl, J., Granroth, G., Miller, R., Stansberry, D., Proffen, T. & Taylor, R. (2014). *IEEE 10th International Conference on e-Science*, Vol. 1, pp. 223–230. Piscataway: IEEE.

[bb33] Squires, G. L. (2012). *Introduction to the Theory of Thermal Neutron Scattering.* Cambridge University Press.

[bb34] Togo, A. & Tanaka, I. (2015). *Scr. Mater.* **108**, 1–5.

[bb35] Trivedi, P. K. & Zimmer, D. M. (2007). *Copula Modeling: an Introduction for Practitioners.* Boston, Delft: Now Publishers.

[bb36] Weber, F., Rosenkranz, S., Pintschovius, L., Castellan, J.-P., Osborn, R., Reichardt, W., Heid, R., Bohnen, K.-P., Goremychkin, E. A., Kreyssig, A., Hradil, K. & Abernathy, D. L. (2012). *Phys. Rev. Lett.* **109**, 057001. 10.1103/PhysRevLett.109.057001 23006199

[bb37] Wu, P., Fan, F.-R., Hagihala, M., Kofu, M., Peng, K., Ishikawa, Y., Lee, S., Honda, T., Yonemura, M., Ikeda, K., Otomo, T., Wang, G., Nakajima, K., Sun, Z. & Kamiyama, T. (2020). *New J. Phys.* **22**, 083083.

