# Supplementary material for: Optimization and inference of bin widths for histogramming inelastic neutron scattering spectra
Source: J Appl Crystallogr. 2022 May 25;55(Pt 3):533–43. doi: 10.1107/S1600576722003624 (PMC9172031; doi:10.1107/S1600576722003624)
Supplement: Supplementary file 1 [file j-55-00533-sup1.pdf]

JOURNAL OF APPLIED CRYSTALLOGRAPHY

Volume 55 (2022)

Supporting information for article:

Optimization and inference of bin widths for histogramming inelastic neutron scattering spectra

Kazuyoshi Tatsumi, Yasuhiro Inamura, Maiko Kofu, Ryoji Kiyanagi and Hideaki Shimazaki

S1. Simulated data based on the first principles calculations for the harmonic phonon states of fcc Cu

In the details of the first principles band calculations of the VASP code, prior to the force constant calculation, we optimized the lattice constant of fcc Cu at 0 K with the Monkhorst & Pack (1976) 5 × 5 × 5 k-point sampling mesh. The cutoff energy for the plane wave basis set was 500 eV, which was the same as in the force constant calculations.

We calculated the following quantity proportional to theoretical DDSCS for the coherent INS (Squires, 2012):

$$\frac{\partial^2\sigma}{\partial\Omega\partial E} \propto \frac{k_f}{k_i}\exp(-q^2\langle u^2\rangle)\sum_{\boldsymbol{\tau}}\sum_{\lambda}\frac{(\boldsymbol{q}\cdot\boldsymbol{e}_\lambda)^2}{\omega_\lambda}\langle n_\lambda + 1\rangle\delta(\omega - \omega_\lambda)\delta(\boldsymbol{q} - \boldsymbol{q}_0 - \boldsymbol{\tau}) \quad (6)$$

with $\boldsymbol{\tau}$ of a reciprocal lattice vector, $\lambda = (\boldsymbol{q}_0, s)$ with a wave vector $\boldsymbol{q}_0$, and a phonon mode index s. ω_λ and $\boldsymbol{e}_\lambda$ are phonon eigenvalue and eigenvector, respectively.

$\langle u^2\rangle = \frac{\hbar}{6mN}\sum_{\lambda'}\frac{\coth(\frac{1}{2}\hbar\omega_{\lambda'}\beta)}{\omega_{\lambda'}}$ for a cubic crystal (Squires, 2012) with the number of wave vectors *N*, atom mass *m*, and inverse temperature *β*. The *β* values were set according to the experimental conditions. $\langle u^2\rangle$ was calculated as the mean square displacement along an arbitrary axis by using an option of the phonon calculation code (Togo & Tanaka, 2015), where the phonon states were sampled on the wave vectors of the 30 × 30 × 30 mesh of the Monkhorst–Pack scheme in the first Brillouin zone of the primitive cell. $\langle n_\lambda\rangle$ is the thermal average of the phonon number.

S2. Formulas for calculating 4D histograms using cumulative sums

We calculated 4D histograms whose bin widths were multiples of the fine bin widths of the initial histogram. The number of possible bin width combinations is $\sim \prod_i N_i$, where N_i is the number of bins along the axis i in the initial histogram. For a histogram whose bin width on the axis i is $n_i \Delta_i^{ini}$ (where Δ_i^{ini} is the corresponding bin width of the initial histogram), $\prod_i n_i$ count sums are needed per bin. The count in a bin of the initial histogram is denoted as $k_{i_1,i_2,i_3,i_4}^{ini}$, where i_k is the bin index on the kth axis. The cumulative sum array element $A_{q,r,s,t}$ of the initial histogram

$$A_{q,r,s,t} = \sum_{i_1=1}^{q} \sum_{i_2=1}^{r} \sum_{i_3=1}^{s} \sum_{i_4=1}^{t} k_{i_1,i_2,i_3,i_4}^{ini} \qquad \text{(S1)}$$

can replace the count sums by a smaller number of the cumulative sum array element sums, decreasing the whole computation. For the histogram of the bin-width set $\{n_i \Delta_i\}$, we introduce an operator p_i that decreases the ith axis index of an array element by n_i, for example,

$$p_1 A_{q,r,s,t} = A_{q-n_1,r,s,t}$$

For the histogram of the bin-width set $\{n_i \Delta_i\}$, all of which n_i are not one, the count of a bin $k_{i,j,h,l}$ is expressed as follows:

$$k_{i,j,h,l} = A_{in_1,jn_2,hn_3,ln_4} + \sum_{m=1}^{4} (-1)^m \left(\sum_{(k_1,\dots,k_m)} p_{k_1} \cdots p_{k_m} \right) A_{in_1,jn_2,hn_3,ln_4} \qquad \text{(S2)}$$

Similar formulas are obtained for the histograms of the other dimensions. We can neglect the sum operation along the corresponding axes when some of the $\{n_i\}$ are equal to one. For the

number of such $\{n_i\}$ as z, we adopted the formula for the 4-z dimensional histograms to decrease the computation.

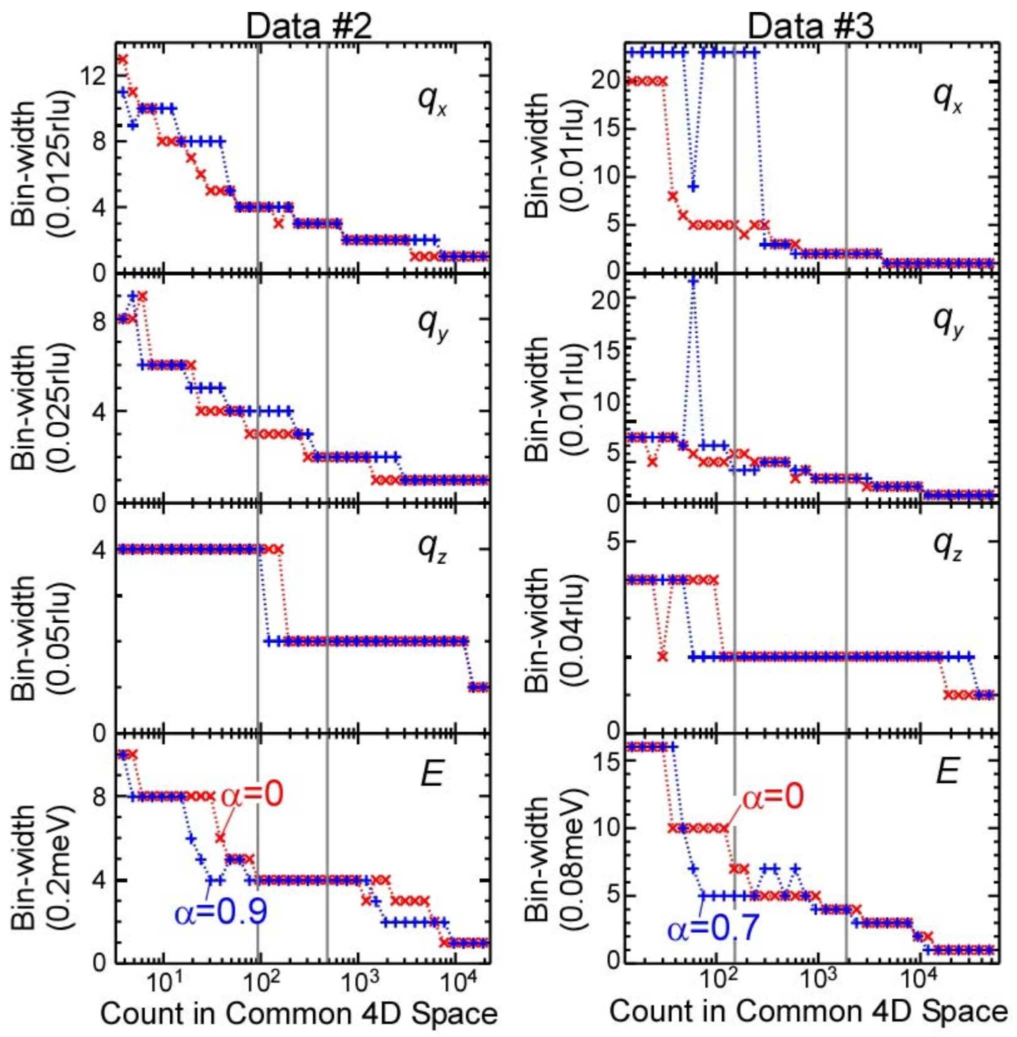

Fig.S1 Tatsumi *et al.*

Figure S1 Count dependencies of the extrapolated optimal bin widths with $\alpha \neq 0$ and = 0 for the experimental Data #2 The vertical gray lines and the units in the vertical axes have the same meanings as those in Fig. 3.

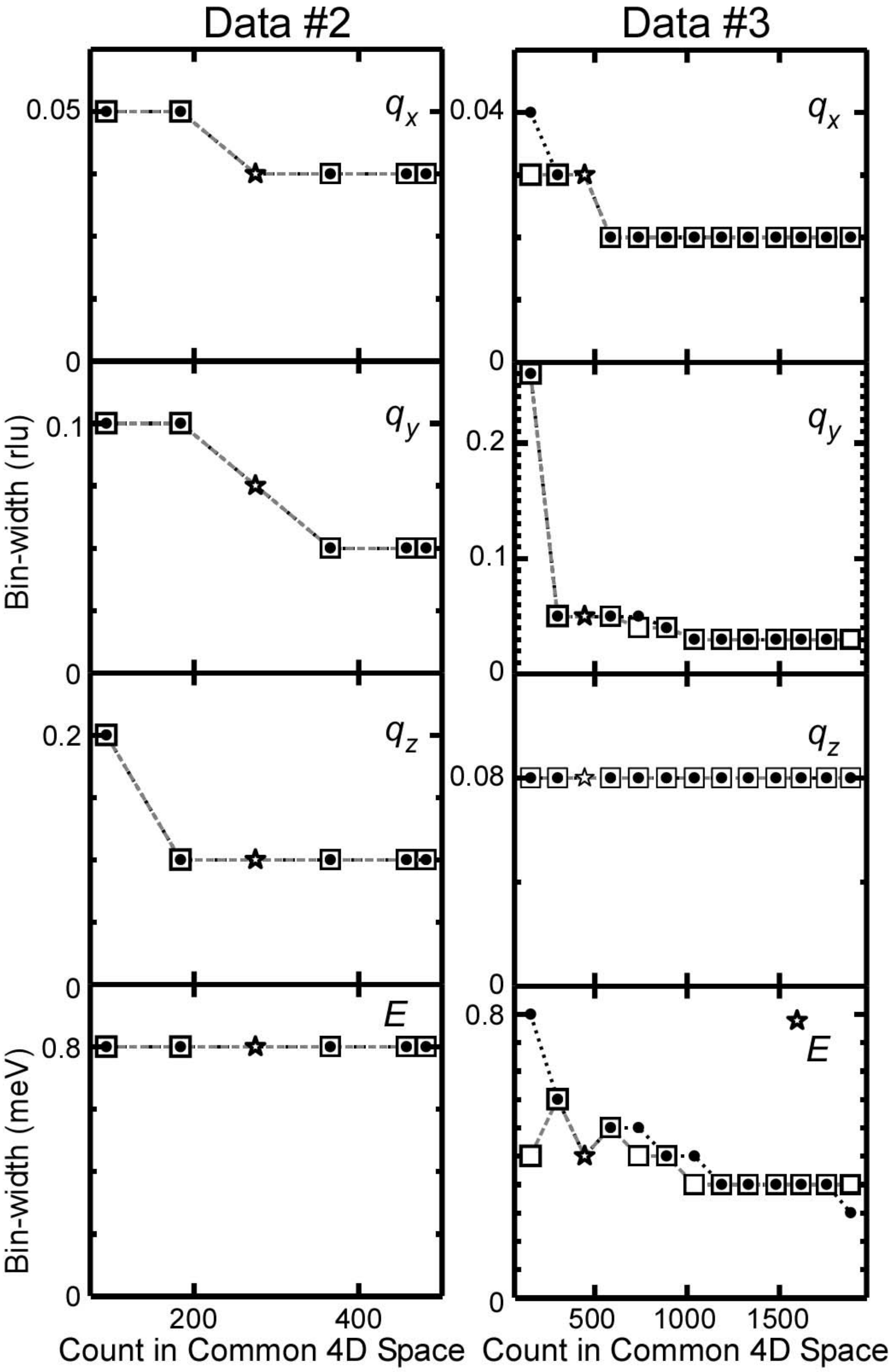

Fig.S2 Tatsumi *et al.*

Figure S2 Comparison of the bin widths optimized by Eq. (2) and inferred by the extrapolation of Eq. (5) for the experimental Data #2 and #3. The minimum tick spacings of the vertical axis have the same meaning as those in Fig. 5.

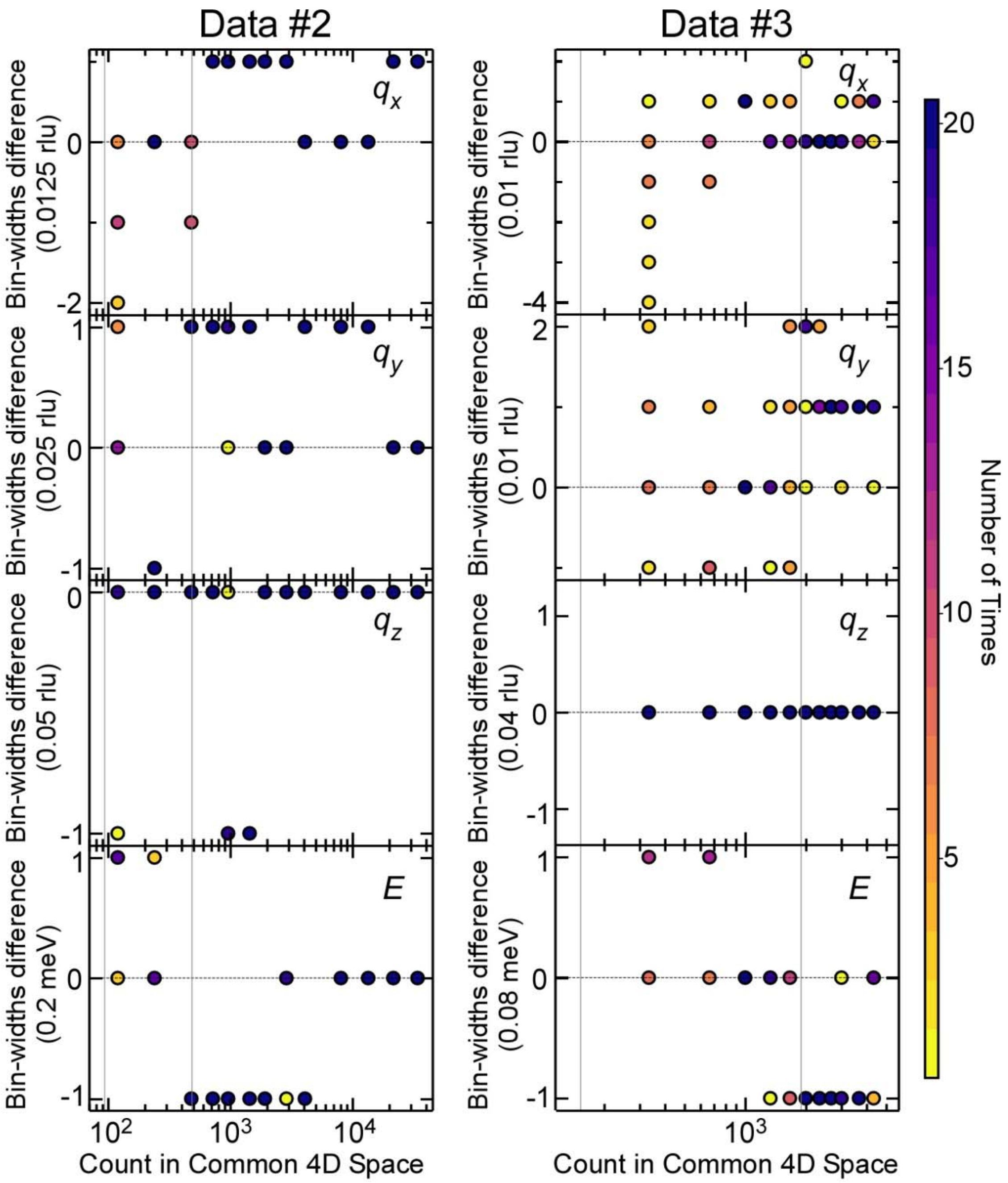

Fig.S3 Tatsumi *et al.*

Figure S3 Convergence of the solutions on the simulated Data #2 and #3. The vertical gray lines, minimum tick spacings of the vertical axis, and color scale have the same meanings as those in Fig. 9.
